# Supplementary material for: Electronic and Nuclear Subsystem Response in Hybrid Halide Perovskites Under γ-Irradiation
Source: Nanomaterials (Basel). 2025 Sep 25;15(19):1474. doi: 10.3390/nano15191474 (PMC12526180; doi:10.3390/nano15191474)
Supplement: Supplementary file 1 [file nanomaterials-15-01474-s001.zip › nanomaterials-3890457-supplementary.pdf]

nanomaterials

MDPI

Supporting information

Electronic and Nuclear Subsystem Response in Hybrid Halide Perovskites Under γ-Irradiation

Ivan E. Novoselov [1,*] and Ivan S. Zhidkov [1,2,3]

1 Institute of Physics and Technology, Ural Federal University, Mira 19 Street, 620002 Yekaterinburg, Russian Federation
2 M.N. Mikheev Institute of Metal Physics of Ural Branch of Russian Academy of Sciences, S. Kovalevskoi 18 Street, 620108 Yekaterinburg, Russian Federation
3 Federal Research Center for Problems of Chemical Physics and Medicinal Chemistry RAS, Semenov ave, 1, 142432, Chernogolovka, Moscow region, Russian Federation
* Correspondence: i.e.novoselov@urfu.ru

Detailed Information about the Modeling Settings

The material definitions in the DetectorConstruction class included atomic composition (charge, atomic mass), elemental fractions, density, and sample geometry.

The PrimaryGeneratorAction class defined source parameters (position, energy, and number of incident particles). Energy deposition was tracked in the SteppingAction class, using GetTotalEnergyDeposit for total absorbed energy and GetNonIonizingEnergyDeposit for non-ionizing losses.

Monte-Carlo simulations were carried out using GEANT4 (v11.3.2) with a custom PhysicsList class based on G4EmStandardPhysicsWVI to assess the electromagnetic processes with particles across a wide energy range (10 eV – 100 MeV) accurately. It includes Rayleigh, Compton, photoelectric, ionization, multiple scattering, pair production, and γ-nuclear processes. For e^-/e^+, multiple scattering was modeled with G4WentzelVIModel combined with single elastic scattering at large angles. Ion ionization was treated using the Lindhard–Sorensen model. The decay processes were treated via G4Decay and G4RadioactiveDecayBase.

Compton scattering was modeled by using the Klein–Nishina model, which reduces to Thomson scattering at low energies and accounts for relativistic effects at high energies. Multiple scattering was described using the G4UrbanMscModel.

In the GammaNuclearPhysics class, γ-nuclear interactions were included with reaction thresholds at Emax1 = 5 MeV and Emax2 = 10 MeV. Depending on energy, either the G4LowGammaNuclearModel or the G4CascadeInterface was applied. This choice reflects the systematics of photonuclear cross-sections: in heavy elements such as Pb and I, the giant dipole resonance (GDR) already develops at ~5–7 MeV, with maximum cross-sections around 10–15 MeV. In contrast, for lighter elements (H, C, N) the onset of photonuclear reactions occurs at higher energies (~15–20 MeV) [1-3]. Thus, including γ-nuclear models from 5 MeV and 10 MeV ensures that the “tails” of the relevant process cross-sections in heavy and light atoms, respectively, in perovskites are properly accounted for.

For e−/e+, multiple scattering was modeled with G4WentzelVIModel combined with single elastic scattering at large angles. Ion ionization was treated using the Lindhard–Sorensen model. The list also specified particle transport, the minimum step size for secondary generation, and decay processes: G4Decay and G4RadioactiveDecayBase.

 https://doi.org/10.3390/nano15191474

For different perovskite thicknesses, production cuts and energy thresholds were adjusted accordingly. For 1 cm absorbers, a production cut of 0.5 mm was applied, with thresholds of 30 keV for γ-rays, 396 keV for electrons, 380 keV for positrons, and 50 keV for secondary protons, which dominate the low-energy range (< 5 MeV). For higher incident energies (> 5 MeV), the cut was increased to 1 mm with thresholds of 47 keV (γ), 659 keV (electrons), 627 keV (positrons), and 100 keV (protons). For thinner films, cuts and thresholds were scaled down proportionally (by roughly an order of magnitude per thickness order).

This choice ensures a physically reasonable balance between computational efficiency and the accurate transport of secondaries within the respective geometry. Importantly, lower thresholds were used in thin layers to resolve localized ionization and vacancy generation, while in thick absorbers coarser cuts adequately capture the macroscopic energy deposition.

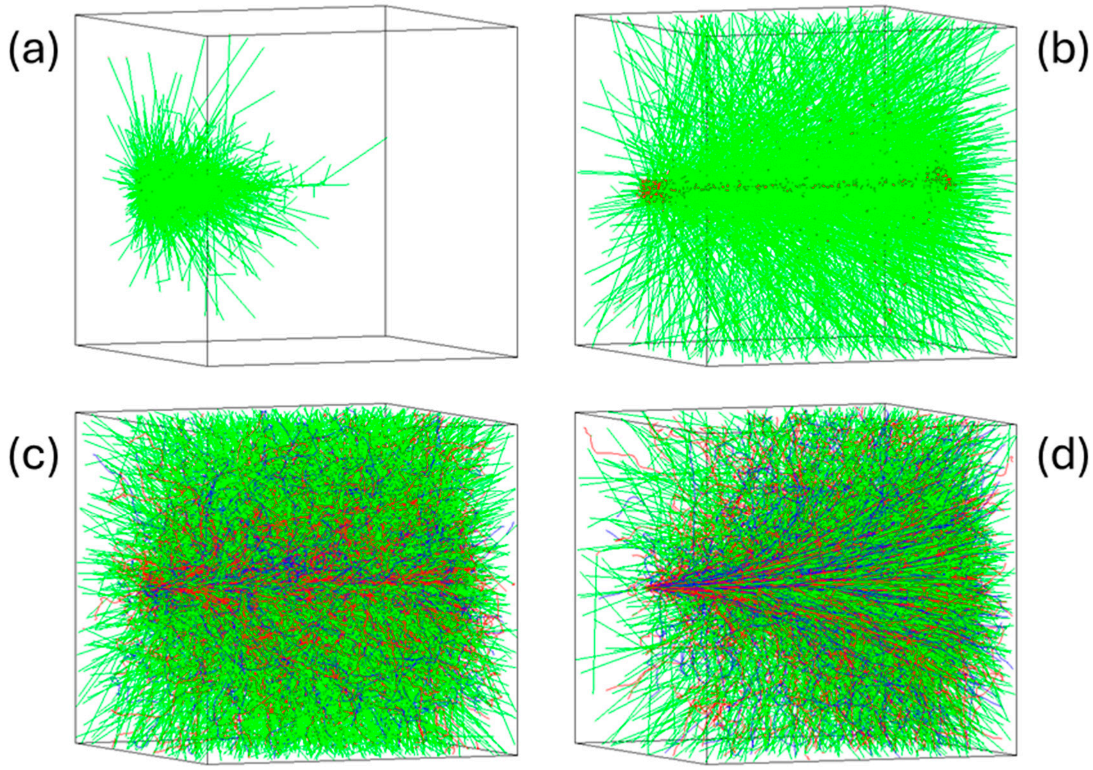

Figure S1. γ-ray flows in simulation volume at various energies (a – 0.1 MeV, b – 1 MeV, c – 10 MeV, d – 90 MeV; green - γ-rays, red – secondary electrons, blue – secondary positrons).

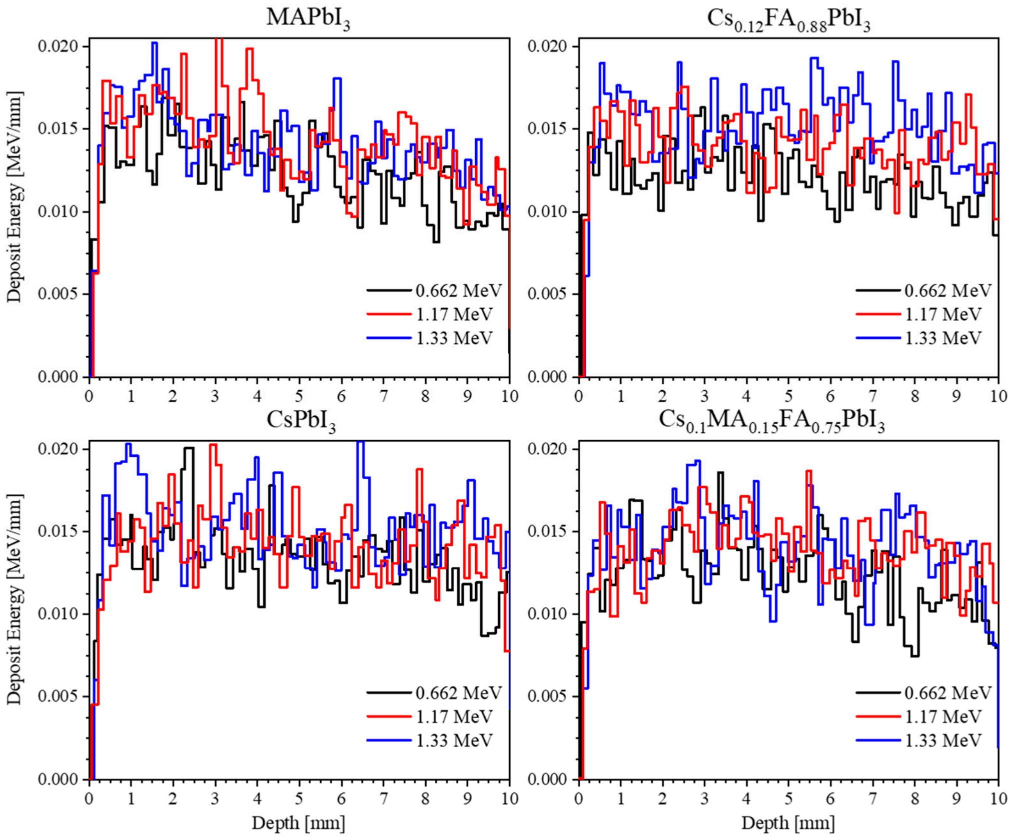

Figure S2. Simulated deposit energy as a function of perovskite thickness for various perovskite materials at common calibration energies.

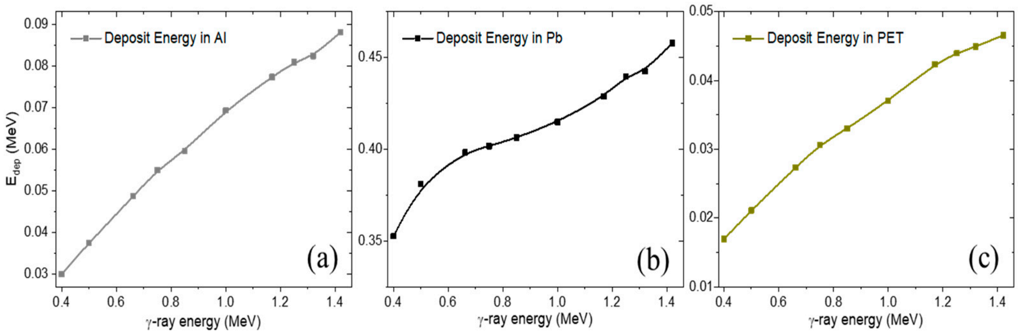

Figure S3. Calculated deposit energy in different reference materials (Al, Pb, PET).

Table S1. Secondary particles generated in MAPbI3 (1 cm) under γ-ray irradiation and their energy distribution.

Incident energy (MeV)	Particle	Number of particles	Mean particle energy (MeV)	Min particle energy (keV)	Max particle energy (MeV)
0.1	electron	102956	0.0321	0.0139	0.0997
	gamma	112	0.0419	30	0.0629
0.2	electron	102258	0.109	0.0506	0.2
	gamma	961	0.056	30	0.159
0.3	electron	77788	0.161	0.0644	0.267

	gamma	1640	0.0671	30	0.244
0.4	electron	59576	0.197	0.0403	0.367
	gamma	1848	0.0744	30	0.341
0.5	electron	49095	0.232	0.0164	0.467
	gamma	1946	0.0834	30	0.435
0.6	electron	41315	0.274	0.116	0.567
	gamma	620	0.168	99	0.447
0.7	electron	37048	0.312	0.138	0.667
	gamma	727	0.181	99.1	0.515
0.8	electron	33653	0.353	0.111	0.767
	gamma	813	0.19	99.1	0.611
0.9	electron	31203	0.396	0.126	0.867
	gamma	923	0.201	99	0.658
1	electron	29282	0.44	0.185	0.967
	gamma	1055	0.216	99	0.798
2	positron	1361	0.482	32	0.874
	electron	21132	0.883	0.116	1.97
	gamma	4938	0.408	99	1.66
3	positron	3353	0.98	4.85	1.97
	electron	20618	1.23	0.118	2.97
	gamma	9960	0.449	99	2.45
4	positron	4999	1.47	0.637	2.98
	electron	21249	1.55	0.163	3.97
	gamma	14139	0.477	99	3.35
5	positron	6407	1.96	0.851	3.98
	electron	22311	1.84	0.182	4.97
	gamma	17871	0.51	99.1	4.18
6	positron	7683	2.46	1.06	4.98
	electron	23425	2.11	0.266	5.97
	gamma	21311	0.533	99	5.33
7	I127	1	0.00015	0.145	0.00015
	Pb206	2	0.00013	0.106	0.00015
	Pb208	2	0.00008	0.0268	0.00014
	positron	8780	2.96	5.17	5.98
	electron	24776	2.4	0.018	6.97
	gamma	24123	0.571	23	6.27
8	I127	5	0.00031	0.186	0.00054
	Pb206	3	0	0.00168	0
	Pb206[2200.160]	3	0.00014	0.0566	0.00022

Continuation of **Table S1.**

Incident energy (MeV)	Particle	Number of particles	Mean particle energy (MeV)	Min particle energy (keV)	Max particle energy (MeV)
8	Pb207	2	0.00028	0.124	0.00043
	Pb208	5	0.00031	0.0907	0.00052
	positron	9772	3.44	6.03	6.98
	electron	25808	2.67	0.117	7.97
	gamma	27070	0.61	32	7.2
9	I127	12	0.00041	0.142	0.00086
	Pb204	1	0.00013	0.129	0.00013
	Pb206	6	0.00031	0.0672	0.00061
	Pb207	4	0.00025	0.00084	0.00048
	Pb207[1633.356]	1	0.00049	0.488	0.00049
	Pb208	12	0.00032	0.0973	0.00054
	positron	10587	3.94	2.14	7.98
	electron	27058	2.9	0.114	8.97
	gamma	30138	0.652	50	8.1
10	I126[110.850]	1	0.00267	2.67	0.00267
	I127	26	0.00052	0.0385	0.00117
	Pb204	2	0.00028	0.143	0.00041
	Pb206	23	0.00059	0.00168	0.00401
	Pb206[2658.320]	3	0.00001	0.00488	0.00001
	Pb206[4027.300]	3	0.00025	0.215	0.00028
	Pb207	11	0.0019	0.0773	0.00453
	Pb208	25	0.00034	0.0816	0.0007
	Te126[666.338]	1	0.00001	0.00944	0.00001
	positron	11369	4.44	7.76	8.98
	electron	28252	3.14	0.11	9.97
	gamma	32410	0.699	57.6	9.01
	neutron	13	0.465	67.6	1.14
	proton	2	0.064	8.47	0.12
20	B11	1	0.108	108	0.108
	Be8	1	3.99	3990	3.99
	C12	3	0.526	59.2	1.33
	I125	54	0.0148	0.98	0.0406
	I126	2	0.0318	30.6	0.0329
	I127	58	0.00351	0.263	0.0091
	Pb204	3	0.0105	1.64	0.0153
	Pb205	3	0.00541	3.22	0.00802
	Pb206	25	0.00644	0.318	0.022
	Pb207	3	0.00212	0.594	0.00516
	Pb208	14	0.00188	0.52	0.00319
	Te126	2	0.00001	0.00189	0.00002
	neutron	148	0.956	35.5	4.86
	proton	10	0.35	7.13	1.35
	positron	16551	9.29	9.78	19
	electron	35593	5.75	0.0629	20
	gamma	50481	1.22	35.5	19

Continuation of **Table S1.**

Incident energy (MeV)	Particle	Number of particles	Mean particle energy (MeV)	Min particle energy (keV)	Max particle energy (MeV)
30	B11	1	0.353	353	0.353
	C12	2	1.01	606	1.42
	I124	6	0.0317	7.89	0.0658
	I125	7	0.0384	13.7	0.0755
	I126	2	0.0208	19	0.0226
	I127	8	0.012	1.43	0.0526
	Pb203	2	0.0348	32.5	0.0371
	Pb204	2	0.00383	3.36	0.00429
	Pb205	4	0.0257	2.46	0.0474
	Pb206	3	0.00382	0.224	0.00646
	Pb207	1	0.00136	1.36	0.00136
	Pb208	7	0.00716	1.66	0.0164
	Te124	6	0.00002	0.00157	0.00004
	Te124[1325.513]	1	0.00001	0.00987	0.00001
	Te124[602.727]	2	0.00002	0.00226	0.00003
	positron	19455	14.2	6.31	29
	electron	39501	8.48	0.105	30
	gamma	61777	1.72	83.5	28.5
	neutron	58	1.68	84.6	5.86
	proton	7	3.25	83.5	12.9
40	Be8	1	5.87	5870	5.87
	C12	1	0.0203	20.3	0.0203
	I123	4	0.0261	11.9	0.0365
	I124	3	0.0947	32	0.18
	I126	1	0.0272	27.2	0.0272
	I127	5	0.00945	0.681	0.0249
	Li7	1	1.31	1310	1.31
	N14	1	0.199	199	0.199
	Pb202	2	0.04	7.9	0.0721
	Pb203	1	0.0243	24.3	0.0243
	Pb204	3	0.0271	6.37	0.0448
	Pb205	1	0.109	109	0.109
	Pb206	1	0.0111	11.1	0.0111
	Pb208	2	0.00727	1.09	0.0134
	Te124	3	0.00002	0.00157	0.00004
	Te124[602.727]	2	0.00002	0.0124	0.00003
	Te125	1	0.113	113	0.113
	Xe126	1	0.00001	0.00573	0.00001
	alpha	4	3.74	2670	5.42
	deuteron	1	12.4	12400	12.4
	positron	21438	19	8.79	39
	electron	42388	11.2	0.0232	40
	gamma	70265	2.26	87.6	38.4
	neutron	56	1.83	84.6	16.9
	proton	6	5.51	33	20.6

Continuation of **Table S1**.

Incident energy (MeV)	Particle	Number of particles	Mean particle energy (MeV)	Min particle energy (keV)	Max particle energy (MeV)
50	B11	1	0.65	650	0.65
	C12	1	0.013	13	0.013
	I122	2	0.0212	0.962	0.0415
	I123	3	0.0638	25.5	0.139
	I124	1	0.254	254	0.254
	I126	3	0.0207	0.0001	0.058
	I126[56.430]	1	0.0092	9.2	0.0092
	I127	5	0.0137	1.24	0.0381
	N14	1	0.0467	46.7	0.0467
	Pb201	2	0.0404	9.46	0.0714
	Pb202	1	0.0365	36.5	0.0365
	Pb203	4	0.0715	26.7	0.166
	Pb205	2	0.0612	0	0.122
	Pb205[2.329]	1	0.00534	5.34	0.00534
	Pb206	1	0.00068	0.682	0.00068
	Pb207	2	0.00278	0.00084	0.00555
	Pb208	3	0.00557	0.398	0.0159
	Te122	2	0.00002	0.0014	0.00004
	Te122[564.094]	1	0.00002	0.0152	0.00002
	Te123	4	0.0359	0.00011	0.144
	positron	22907	23.8	9.14	49
	electron	44520	13.8	0.107	50
	gamma	77575	2.76	23.6	48.2
	neutron	74	2.26	72.8	22.7
	proton	6	4.18	151	9.25
60	B10	1	0.315	315	0.315
	C12	1	0.0124	12.4	0.0124
	Hg200[367.943]	2	0.00001	0.0009	0.00001
	I122	1	0.0433	43.3	0.0433
	I123	1	0.0815	81.5	0.0815
	I125	1	0.0679	67.9	0.0679
	I126	3	0.0113	0.0001	0.0245
	I126[56.430]	1	0.0148	14.8	0.0148
	I127	10	0.0151	0.252	0.0533
	Pb200	2	0.0349	4.95	0.0648
	Pb201	1	0.0329	32.9	0.0329
	Pb202	3	0.0404	27.7	0.0607
	Pb204	2	0.0912	37.5	0.145
	Pb206	1	0.00076	0.76	0.00076
	Te122	3	0.051	0.0014	0.0964
	Te122[564.094]	1	0.00002	0.0239	0.00002
	Te126	2	0.00001	0.00189	0.00002
	Tl201[331.170]	1	0.00001	0.00666	0.00001
	positron	24103	28.6	13.8	58.9
	electron	46588	16.4	0.104	60
	gamma	83881	3.25	80.3	55.3

Continuation of **Table S1.**

Incident energy (MeV)	Particle	Number of particles	Mean particle energy (MeV)	Min particle energy (keV)	Max particle energy (MeV)
60	neutron	68	2.85	21.5	28.3
	proton	8	3.79	18.2	10.1
70	Hg200[367.943]	2	0.00001	0.0117	0.00001
	I121	2	0.0745	3.07	0.146
	I123	1	0.178	178	0.178
	I125	2	0.0453	28.4	0.0621
	I126	3	0.0297	5.15	0.0713
	I127	6	0.0175	0.741	0.0744
	Pb199	1	0.0544	54.4	0.0544
	Pb200	2	0.133	68.3	0.199
	Pb202	2	0.079	69.8	0.0881
	Pb203	1	0.0353	35.3	0.0353
	Pb204	2	0.142	105	0.18
	Pb205	1	0.00642	6.42	0.00642
	Pb207	1	0.00527	5.27	0.00527
	Pb208	1	0.00077	0.77	0.00077
	Te121	3	0.0916	0.00096	0.275
	Te121[212.191]	2	0.00002	0.0194	0.00002
	alpha	3	5.07	710	8.56
	positron	25139	33.3	16.3	68.9
	electron	48274	18.9	0.147	70
	gamma	89372	3.74	54.6	64.6
	neutron	78	3.37	66.5	37.3
	proton	7	6.33	56.4	29.4
80	Hg200[367.943]	4	0.00001	0.0039	0.00001
	I123	1	0.337	337	0.337
	I125	3	0.0687	15	0.14
	I126	8	0.0232	0.00005	0.0813
	I126[110.850]	1	0.0153	15.3	0.0153
	I126[122.170]	2	0.0021	0.00006	0.00421
	I126[237.230]	1	0.00164	1.64	0.00164
	I127	10	0.0253	0.296	0.0985
	Pb200	4	0.122	59.8	0.214
	Pb203	1	0.036	36	0.036
	Pb204	1	0.144	144	0.144
	Pb206	2	0.00218	1.97	0.00238
	Pb207	2	0.0124	4.63	0.0202
	Pb208	5	0.00478	0.288	0.0201
	Te121	1	0.151	151	0.151
	Te126	4	0.00001	0.00189	0.00002
	Te126[666.338]	2	0.00001	0.00944	0.00001
	Tl201	1	0.135	135	0.135
	positron	25938	38	26.9	78.9
	electron	49492	21.5	0.144	80
	gamma	94166	4.22	73.3	74
	neutron	72	3.44	78.1	43.3

Continuation of **Table S1**.

Incident energy (MeV)	Particle	Number of particles	Mean particle energy (MeV)	Min particle energy (keV)	Max particle energy (MeV)
80	proton	8	6.24	0.934	35.6
90	C12	1	0.634	634	0.634
	I125	5	0.0564	0.715	0.134
	I126	6	0.0641	0.00006	0.178
	I126[237.230]	1	0.00203	2.03	0.00203
	I127	6	0.00551	0.652	0.0249
	Pb198	1	0.24	240	0.24
	Pb199	1	0.0989	98.9	0.0989
	Pb200	1	0.088	88	0.088
	Pb203	1	0.11	110	0.11
	Pb207	3	0.00217	0.00084	0.00332
	Pb207[1633.356]	1	0.00386	3.86	0.00386
	Pb208	4	0.0142	0.496	0.0542
	Te126	3	0.00001	0.00189	0.00002
	Tl200	2	0.0901	0.00041	0.18
	Xe126	4	0.00349	0.00064	0.014
	positron	26619	42.5	17.5	88.9
	electron	50919	24	0.0503	90
	gamma	98682	4.68	35.5	85.7
	neutron	55	2.89	71.7	35.3
	proton	4	10.5	35	40.5

Table S2. Secondary particles emerging from $MAPbI_3$ (1 cm) under γ-ray irradiation and their energy distribution.

Incident energy (MeV)	Particle	Number of particles	Mean particle energy (MeV)	Min particle energy (keV)	Max particle energy (MeV)
0.1	electron	201	0.0455	4.47	0.0668
	gamma	942	0.0841	35.4	0.1
0.2	electron	550	0.101	3.63	0.167
	gamma	16402	0.187	30.3	0.2
0.3	electron	796	0.162	8.39	0.267
	gamma	49629	0.282	31.9	0.3
0.4	electron	811	0.213	8.87	0.365
	gamma	70017	0.374	30	0.4
0.5	electron	882	0.254	10.4	0.467
	gamma	80274	0.465	30.3	0.5
0.6	electron	978	0.306	25.1	0.567
	gamma	85901	0.556	99.8	0.6
0.7	electron	1063	0.346	15.3	0.666
	gamma	89252	0.647	76.8	0.7
0.8	electron	1113	0.394	23.3	0.763
	gamma	91605	0.738	89.6	0.8
0.9	electron	1172	0.432	22.5	0.86
	gamma	93205	0.829	97.6	0.9

Continuation of **Table S2.**

Incident energy (MeV)	Particle	Number of particles	Mean particle energy (MeV)	Min particle energy (keV)	Max particle energy (MeV)
1	electron	1289	0.476	25.3	0.967
	gamma	94359	0.921	99	1
2	positron	72	0.43	80.4	0.772
	electron	2093	0.965	41.8	1.97
	gamma	99956	1.81	93.5	2
3	positron	391	0.893	50.5	1.82
	electron	2945	1.4	59.1	2.96
	gamma	102768	2.65	86.6	3
4	positron	939	1.28	58.2	2.91
	electron	3751	1.83	34.1	3.95
	gamma	104766	3.45	83.5	4
5	positron	1487	1.7	29.8	3.81
	electron	4603	2.24	19.4	4.92
	gamma	106523	4.21	84.2	5
6	positron	2277	2.1	4.75	4.83
	electron	5255	2.64	41.2	5.86
	gamma	108022	4.95	75.8	6
7	positron	3076	2.49	16.4	5.86
	electron	6420	3.09	20.8	6.94
	gamma	109169	5.67	82.1	7
8	positron	3932	2.85	43	6.67
	electron	7186	3.46	33.7	7.91
	gamma	110643	6.36	69.5	8
9	positron	4585	3.27	27.9	7.66
	electron	7804	3.8	24.7	8.9
	gamma	112135	7.03	80.3	9
10	positron	5375	3.64	36.9	8.97
	electron	8548	4.2	11	9.86
	gamma	113094	7.7	78.1	10
	neutron	13	0.455	35.2	1.14
20	positron	11516	7.4	55.9	18.8
	electron	14383	7.88	9.91	19.7
	gamma	123039	13.7	75.3	20
	neutron	147	0.942	13.6	4.86
30	positron	15254	11.4	103	28.8
	electron	18068	11.7	20.5	29.8
	gamma	130315	19	73.3	30
	neutron	57	1.59	1.17	5.86
40	positron	17864	15.2	88.7	38.7
	electron	20496	15.4	12.9	39.9
	gamma	136073	24	78	40
	neutron	56	1.72	84.6	16.9
	proton	1	6.56	6560	6.56
50	positron	19685	19.1	79.2	48.7
	electron	22282	19	10.7	49.6

Continuation of **Table S2.**

Incident energy (MeV)	Particle	Number of particles	Mean particle energy (MeV)	Min particle energy (keV)	Max particle energy (MeV)
50	gamma	141317	28.6	55	50
	neutron	73	2.26	72.8	22.7
60	positron	21186	23	98.3	58.1
	electron	23799	22.6	16.1	59.7
	gamma	145860	32.9	83.3	60
	neutron	68	2.81	3.29	28.3
70	positron	22406	26.8	83	68.5
	electron	25133	26	32.6	69.9
	gamma	149859	37.1	82.8	70
	neutron	76	3.38	10.2	37.3
80	positron	23390	30.5	41.5	78.6
	electron	26176	29.4	5.83	79.6
	gamma	153566	41.2	75.1	80
	neutron	70	3.38	12.5	43.3
	proton	1	12.8	12800	12.8
90	positron	24168	34.2	51.2	88.6
	electron	26986	33	24.3	89.6
	gamma	156934	45.2	75.8	90
	neutron	55	2.85	20.2	35.3

References

1. Lewis, F.H.; Walecka, J.D. Electromagnetic Structure of the Giant Dipole Resonance. *Physical Review* **1964**, *133*, B849–B868, doi:10.1103/PhysRev.133.B849.
2. Maruhn, J.A.; Reinhard, P.G.; Stevenson, P.D.; Stone, J.R.; Strayer, M.R. Dipole Giant Resonances in Deformed Heavy Nuclei. *Phys Rev C* **2005**, *71*, 64328, doi:10.1103/PhysRevC.71.064328.
3. Berman, B.L.; Fultz, S.C. Measurements of the Giant Dipole Resonance with Monoenergetic Photons. *Rev Mod Phys* **1975**, *47*, 713–761, doi:10.1103/RevModPhys.47.713.
